# The effects of walking aids on shoulder joint kinematics in older persons: an initial study

**DOI:** 10.1186/s12877-023-04439-3

**Published:** 2023-11-15

**Authors:** Jiemeng Yang, Zhongjun Mo, Yanyu Zhang, Run Ji, Chunjing Tao, Yubo Fan

**Affiliations:** 1 Key Laboratory for Biomechanics and Mechanobiology of Ministry of Education, Beijing Advanced Innovation Centre for Biomedical Engineering, School of Biological Science and Medical Engineering, and with the School of Engineering Medicine, Beihang University, Beijing, 100191 China; 2 Key Laboratory of Human Motion Analysis and Rehabilitation Technology of the Ministry of Civil Affairs, Beijing Key Laboratory of Rehabilitation Technical Aids for Old-Age Disability, National Research Centre for Rehabilitation Technical Aids, Beijing, 100176 China

**Keywords:** Walking aids, Older individuals, Shoulder Joint, Kinematics

## Abstract

**Background:**

Many older persons with degenerative physical functions use walking aids to improve their ambulation ability. The aim of this study was to investigate the effects of walking aids with different configurations on shoulder joint motion in older persons.

**Methods:**

The 3D motion capture system VICON was applied to collect data on gait parameters and shoulder motion characteristics of 6 older persons walking either independently or with the assistance of a footed walking frame and a wheeled walking frame. The different effects of walking aids on gait parameters and the shoulder joint motion of older individuals were quantitatively analyzed.

**Results:**

The gait parameters of the older individuals changed significantly when they used walking frames to assist walking. Compared to independent walking, the range of motion of the shoulder joint was reduced by 79.92% in flexion when walking with a wheeled walking frame. Meanwhile, the range of motion in flexion, extension, and external rotation increased by 76.04%, 85.55%, and 110.99%, respectively, when walking with a footed walking frame.

**Conclusion:**

The motion characteristics of shoulder joints in older persons were significantly affected by using different walking aids. These changes in shoulder joint motion characteristics will lead to potential diseases related to the shoulder musculoskeletal system. These findings are beneficial to determine a walking aid for older people.

## Background

For decades, the rapid growth of the aging population is a worldwide social problem. With the progress of aging, the physiological functions of older people gradually degenerate, especially the degradation of their walking ability which has become one of the most important factors affecting their quality of life [1–3]. For example, the muscle strength of the lower limbs decreased by about 40% compared to adults in their 30 s, which would result in causing body balance function and decreasing their walking ability [4].

The use of walking aids can assist older people in walking, hereby improving cardiopulmonary function, and demonstrating positive significance for promoting the human physiological system and delaying the degradation of physiology functions [5–7]. Walking aids are divided into powered walking aids, functional electrical stimulation walking aids and non-powered walking aids according to the source of the driving force. Non-powered walking aids operated by both arms, such as footed walking frames or wheeled walkers, provide a stable support structure with four legs and are widely used among people in need [5, 8].

However, using walking frames can help older persons to reduce the load on the lower limbs at the cost of increasing the load on the upper limbs, which may increase the risk of potential diseases in the relative parts. Previous research showed that long-term use of walking frames can easily cause upper limb disorders, such as carpal tunnel syndrome, median neuropathy, stress fractures, and upper limb pain [9, 10]. When using a footed walking frame or a wheeled walker, the upper limbs must be moved symmetrically and together because of the construction of the frame and the confinement of motion in the sagittal plane [11]. The study conducted by Simoneau G. [12] showed that the individuals needed to lift the standard walking frame with upper limbs to make it move forward, and the joint moment produced at the shoulder during ambulation with the walking frame is of significant magnitude. The motion angle of the shoulder joint was the largest, followed by the elbow and wrist [13]. The study exerted that the joint range of motion of both upper limbs was absent or decreased when walking with a wheeled walker [14]. Basing the data of seven healthy young individuals using a walking frame, it was found that there were significant differences in shoulder joint moment under different weight-bearing states of the lower limbs [15]. It was pointed out that the activity of shoulder muscles increased to bear the load of lower limbs transfer and maintain balance in paraplegic patients who used a walking aid [16]. Other studies also attempted to investigate the biomechanical effects of different configurations of walking aids, while their focuses were mainly on the gait parameters, effects of balance ability, physiological energy cost or the kinematic and kinetics analyses of lower limbs [17–20]. However, the different effects of the motion characteristics on the upper limbs between the wheeled walking frame and footed walking frame have not been compared and described clearly.

Understanding the effects of different configurations of walking frames on the shoulder joints can help to analyze compensatory responses and injury risks of the upper limbs induced by using walking frames and to suggest an appropriate aid for older people. In this study, the motion data of a group of older individuals in normal walking and aids assisting walking were collected and analyzed. Two kinds of two-arm-operated walking aids including the wheeled walking frame and the footed walking frame were comparatively analyzed. This quantitative investigation focused on the different effects of walking aids on the motion characteristics of the shoulder joints of older individuals.

## Methods

### Experimental materials

Six healthy older individuals, including 4 males and 2 females, with an age of 65.00 ± 3.58 years, an average height of 1.63 ± 0.09 m, a weight of 68.41 ± 4.17 kg, and an average arm length of 0.51 ± 0.02 m, signed an informed consent form and participated in this experiment. All participants had no obvious visual or auditory impairment, or related medical history that had impacts on daily activities and their hands could perform the manipulation of walking aids autonomously. In addition, the participants can walk more than 10 m independently, without assistive devices.

**Fig. 1:**
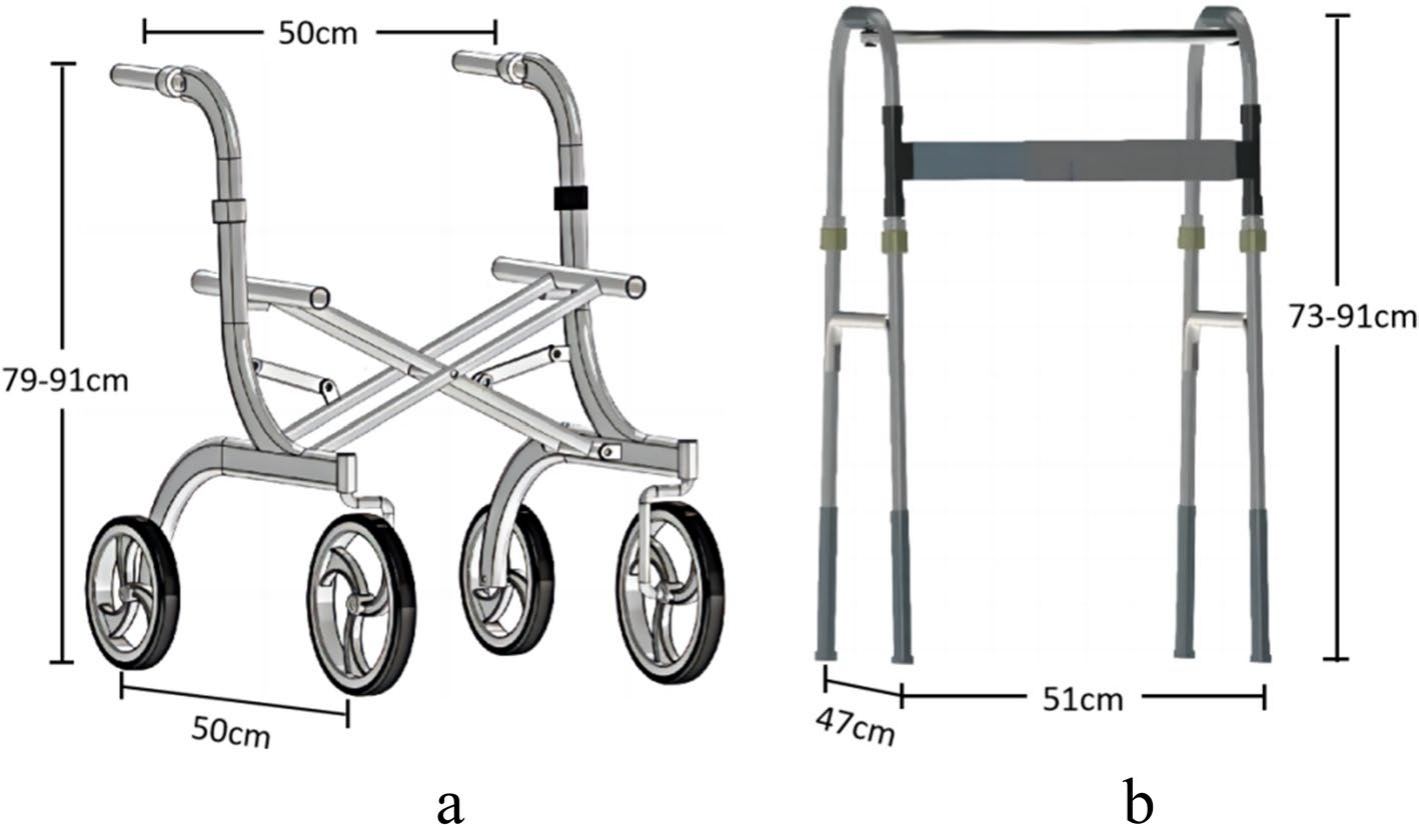
Two configurations of walking frames employed in the experiment. **a** Wheeled walker; **b** Footed walking frame

The two-arm-operated walking frames employed in this experiment were a wheeled walker (FZK-3104, Fuzikon, Foshan, China) and a footed walking frame (YC8202, Fushide, Zhongshan, China), as shown in Fig. 1. The weights of wheeled walker and footed walking frame are 8 kg and 2.55 kg respectively.

### Experimental methods

**Fig. 2:**
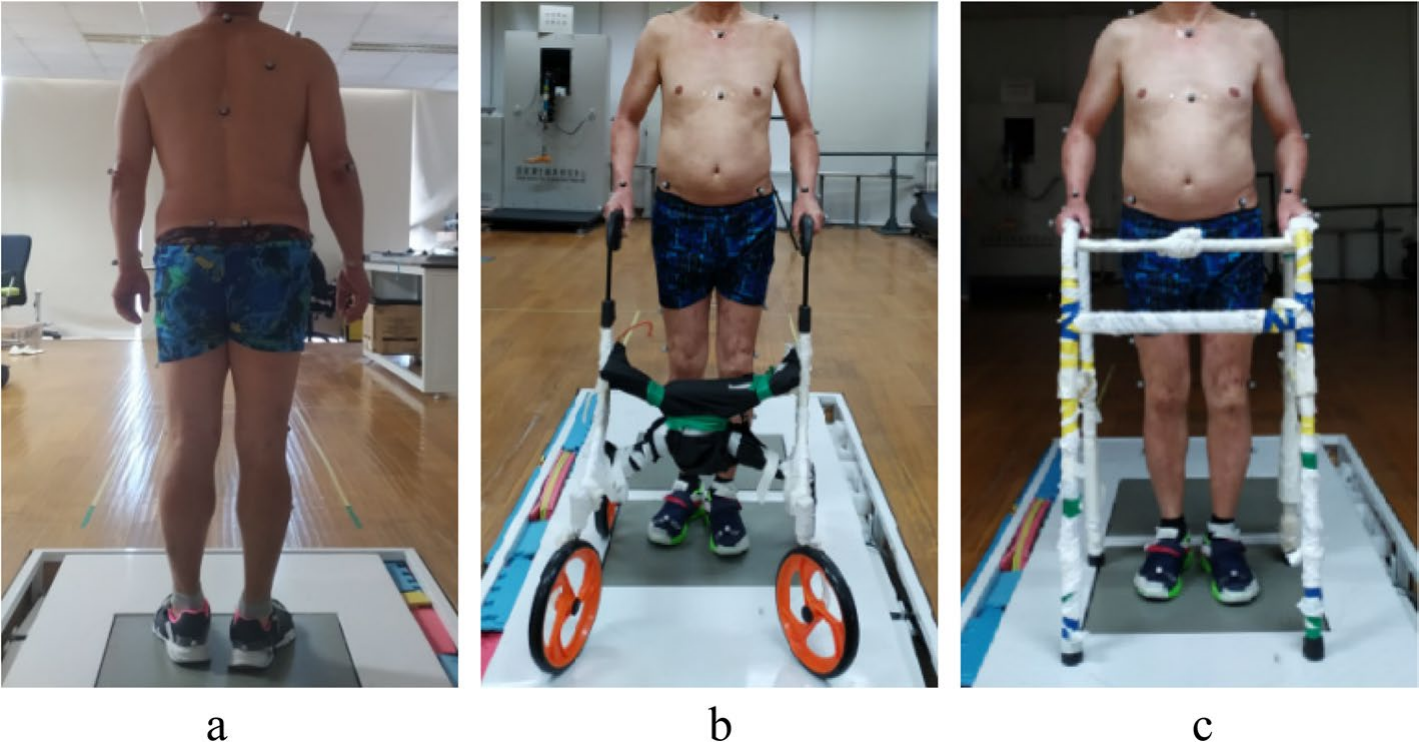
Three tasks in the experiment. **a** Independent walking. **b** Walking with a wheeled walker. **c** Walking with a footed walking frame

Before each experiment, the basic physiological parameters were measured and the height of the wheeled walker and the footed walking frame were adjusted based on the height of the participant’s ulnar styloid process [21, 22]. All participants were trained to walk on the walking path with two configurations of walking frames in the gait laboratory. When using a wheeled walker, the user needed to push it forward. While using a footed walking frame, the user needed to lift it off the ground and place it at a comfortable distance [5, 9, 10]. Until the participants could use the walking frames proficiently, the experiment officially started. Three experimental tasks were carried out, including 1) walking independently through a horizontal walkway, 2) walking through a horizontal walkway with a wheeled walker, and 3) walking through a horizontal walkway with a footed walking frame, as shown in Fig. 2. The length of the walking path was 5 m to cover at least two complete gait cycles. Each task was repeated at least 6 times for each participant to make sure 6 groups of valid data were recorded. The average value of 6 repeated measurements were calculated, and then the average value of six older individuals were used to calculate the mean gait parameters. During the experiment, the order of tasks was random. Participants completed several tasks at a normal pace and were allowed to rest between each task.

**Fig. 3:**
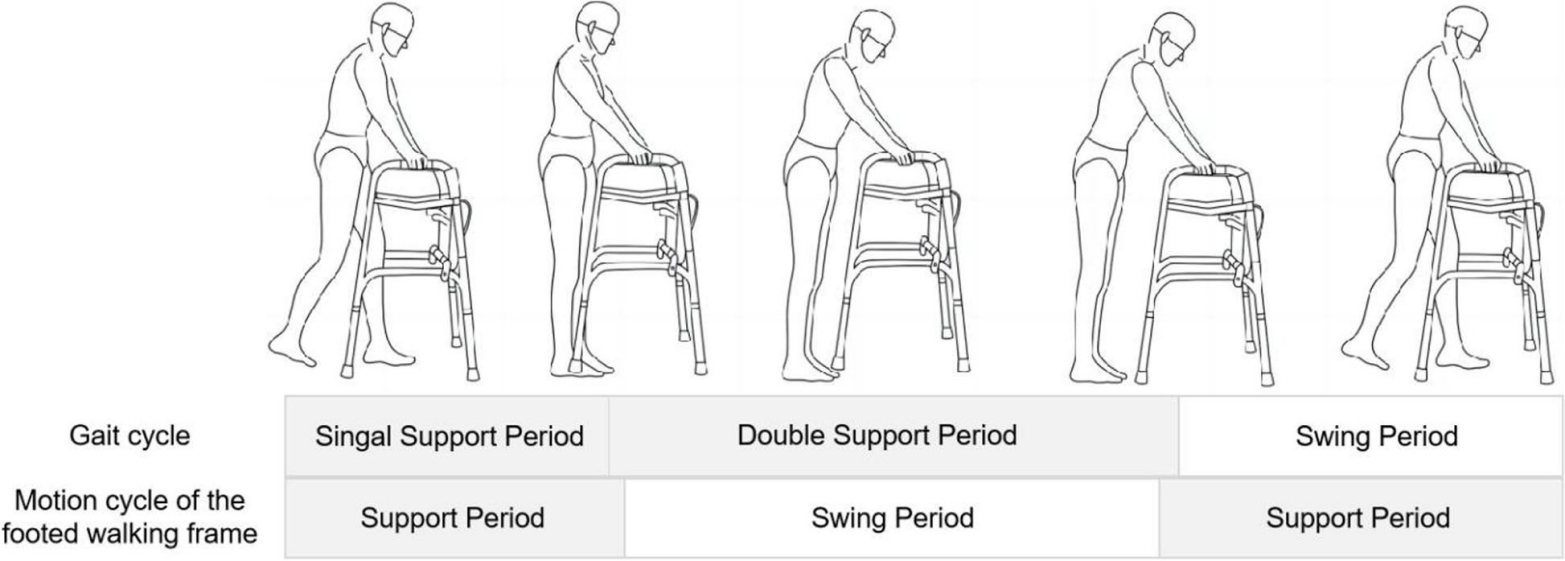
Gait cycle of older individuals and the motion cycle of the footed walking frame

The 3D motion capture system (VICON, Oxford Metrics Limited, UK) was employed to achieve the motion data of all participants. This system was able to reconstruct body segments by capturing the position of anatomical markers with optical cameras ( 8 Vicon Vantage V5 Cameras) and then the kinematics of the joints of the corresponding segments were evaluated by the software (Vicon Nexus 1.8). According to the Plug-in-Gait full body model protocol, a total of 39 markers (14 mm in diameter) were fixed on the participant's head (4 markers), torso (5 markers), upper limbs (7 markers on the left upper limb, 7 markers on the right upper limb), pelvis (4 markers) and lower limbs (6 markers on the left lower limb and 6 markers on the right lower limb). The position of the pelvis was determined by marking the left and right anterior superior iliac spine (ASIS) and the left and right posterior superior iliac spine (PSIS) on the surface of the body. VICON collected the marker coordinates of participants at the same frequency (f = 100 Hz), and the data processing module was used to perform system calibration, data acquisition and data output. The spatio-temporal parameters such as cadence, walking speed, step length, step time, stride length, stride time, single support period, double support period, and the motion data of joints were calculated by VICON Nexus. In this experiment, the gait cycle and the motion cycle of the footed walking frame were divided as shown in Fig. 3. The gait cycle was from the left heel strike to the left heel strike again. The support period of left foot was from the left heel strike to left toe off, and the swing period of left foot was from the left toe off to the left heel strike again [23]. The support period of the footed walking frame was divided through the motion of wrist, because of the height of wrist would not change obviously during the support period of the footed walking frame. The gait parameters and motion data in one gait cycle were processed and analyzed. The joint angle − gait cycle relationship curves were obtained by normalizing and calculating the mean of the 6 older persons by MATLAB R2018b software. Descriptive data were expressed as mean ± standard deviation. The differences in the average data obtained among the three patterns of walking were analyzed with SPSS 23.0, the level of statistical significance was set at 0.05 [14]. The normality test and homogeneity of variances test were conducted at first. If the data satisfied both conditions simultaneously, the ANOVA analysis was performed, otherwise the Nonparametric test was performed.

## Results

### Gait parameters

Six gait parameters of older individuals with ANOVA analysis were displayed in Table 1 and two gait parameters of older individuals with Nonparametric Test were displayed in Table 2. The statistical analysis revealed that there were significant differences in the gait parameters of the participants when they completed different tasks. Compared with independent walking, it produced significant decreases in cadence by walking with two configurations walking aids. And the step time, stride time, and double support period increased significantly when using the footed walking frame, whereas the step length, stride length, walking speed decreased significantly. Compared with using the wheeled walker, the footed walking frame had more significant effects on older individuals, such as increases of step time, stride time, and decreases of cadence, stride length, and single support period.

**Table 1:** The effects of different walking frames on gait parameters with ANOVA analysis

Gait cycle parameters	Independent walking (Pattern 1)	Walking with a wheeled walker (Pattern 2)	Walking with a footed walking frame (Pattern 3)	F	P	Post hoc analysis
Cadence (steps/min)	109.36 ± 7.02	95.98 ± 7.72	52.78 ± 4.49	121.906	0.000	Pattern 1 > Pattern 2** Pattern 1 > Pattern 3*** Pattern 2 > Pattern 3***
Step length (m)	0.58 ± 0.03	0.56 ± 0.04	0.50 ± 0.21	4.029	0.040	Pattern 1 > Pattern 3*
Step time (s)	0.56 ± 0.04	0.62 ± 0.05	1.69 ± 0.16	242.913	0.000	Pattern 1 < Pattern 3*** Pattern 2 < Pattern 3***
Stride length (m)	1.15 ± 0.08	1.12 ± 0.08	0.55 ± 0.08	103.730	0.000	Pattern 1 > Pattern 3*** Pattern 2 > Pattern 3***
Stride time (s)	1.10 ± 0.07	1.26 ± 0.10	2.30 ± 0.21	131.915	0.000	Pattern 1 < Pattern 3*** Pattern 2 < Pattern 3***
Single support period (s)	0.37 ± 0.03	0.43 ± 0.04	0.33 ± 0.05	9.451	0.002	Pattern 2 > Pattern 3**

Statistical significance: * *p* < 0.05, ** *p* < 0.01, *** *p* < 0.001

**Table 2:** The effects of different walking frames on gait parameters with Nonparametric Test

Gait cycle parameters	Independent walking (Pattern 1)	Walking with a wheeled walker ( Pattern 2)	Walking with a footed walking frame ( Pattern 3)	H	P	Post hoc analysis
Walking speed (m/s)	1.05 (1.00,1.10)	0.86 (0.83,0.91)	0.24 (0.21,0.26)	13.053	0.001	Pattern 1 > Pattern 3***
Double support period (s)	0.35 (0.33,0.36)	0.42 (0.37,0.45)	1.58 (1.40,1.69)	14.000	0.001	Pattern 1 < Pattern 3***

Statistical significance: * *p* < 0.05, ** *p* < 0.01, *** *p* < 0.001

### Motion characteristics of pelvis

**Fig. 4:**
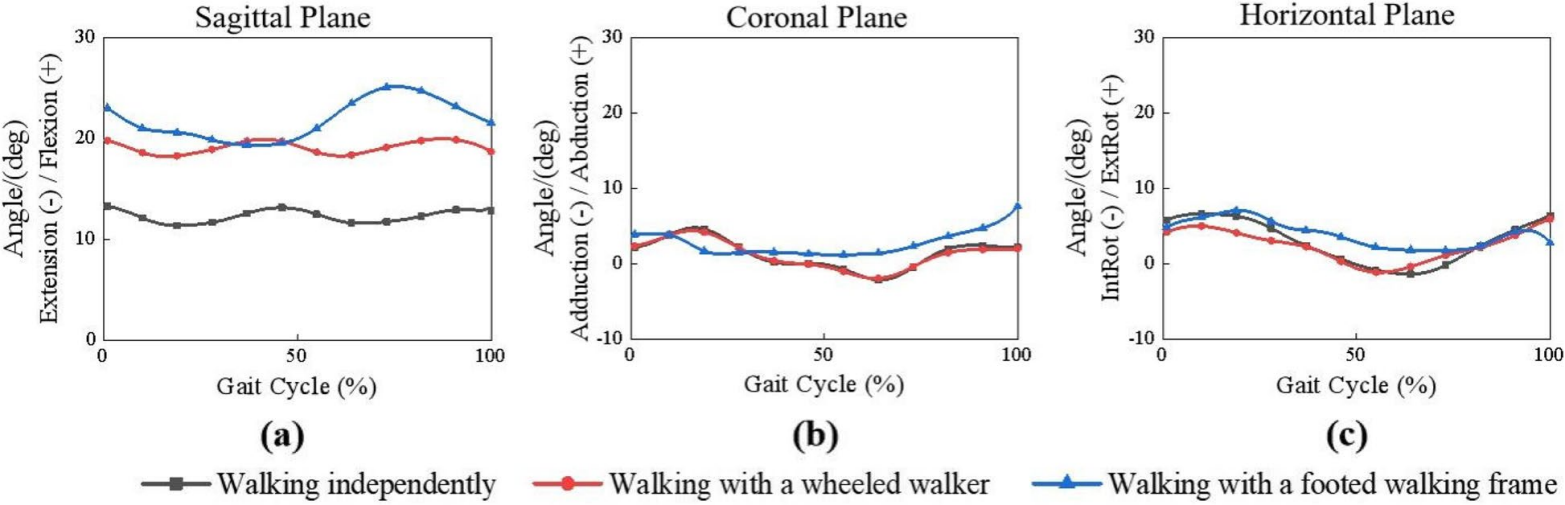
Comparison of the motion angles of the pelvis

The rotational angle-gait cycle curves of the pelvis in sagittal, coronal, and horizontal planes in the different tasks were listed in Fig. 4. When walking independently, the peak of the anteversion angle of the pelvis in the sagittal plane reached 13.15°, which was similar with the results of Murray et al. [24]. Walking with both aids caused additional anteversion of the pelvis in the sagittal plane in the individuals. When walking with the wheeled walker, the rotational angle was greater than independent walking with a peak inclination of 19.98°. When walking using the walking frame, the pelvic anteversion angle varied with the distance between the body and the walking frame, which reached a peak value of 25.18° at the end of the support period. The pelvic rotational angles were inapparent in the coronal plane and horizontal plane.

### Motion characteristics of the shoulder joint

**Fig. 5:**
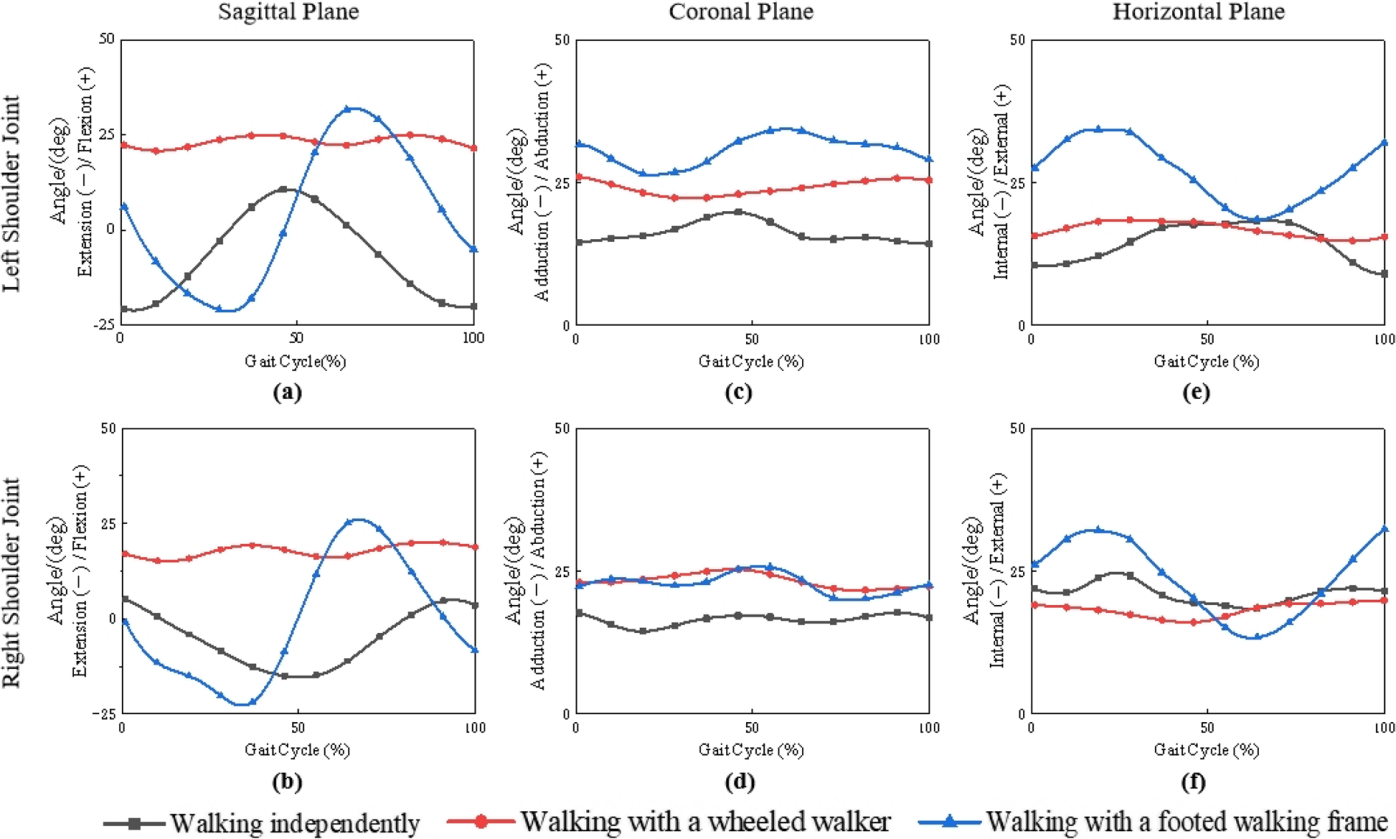
Comparison of the motion angles of the shoulder joints

The rotational angles of shoulder joints in the sagittal, coronal and horizontal planes in a whole gait cycle when the participants did the three tasks subsequently were shown in Fig. 5.

The rotational angles of the left and right shoulder joints in the sagittal plane were shown in Fig. 5(a) and (b), respectively. When walking independently, the arms swung backward with a peak extension angle of 21.07° and forward with a peak flexion angle of 10.70° alternately. When the wheeled walker was used, the rotational direction of the shoulder joints on both sides were the same, and the flexion angle was relatively stable (17.95°-22.55°), while the peak flexion angle was approximately 12.39° higher than that of independent walking. When a footed walking frame was used to assist walking, the rotational angle of the shoulder joints on both sides of the support period was characterized first by extension backward with a peak angle of 22.21°. With lifting the walking frame forward, the shoulder joints were flexed forward with a peak angle of 29.73°. Since the start of the swing period, as the distance between the body and the walking frame decreased gradually, the flexion angle of the shoulder joint decreased gradually to -6.64°, that is, the shoulder joint was slightly extended backward.

The rotation characteristics of the left and right shoulder joints in the coronal plane were shown in Fig. 5(c) and (d). It was found that the participants had a certain degree of abduction on both sides of the shoulder joints in three tasks. The abduction angle of the shoulder joint was the smallest when the participants walked independently with an average abduction angle of 16.32° on the left sides and 16.43° on the right sides, respectively. When using the wheeled walker, the average abduction angle of the left shoulder joint was 24.07°, and that of the right shoulder joint was 23.32°. When the participants walked using the walking frame, the average abduction angle of the left shoulder joint was 30.40°, and that of the right shoulder joint was 22.82°.

The rotational characteristics of the left and right shoulder joints in the horizontal plane were shown in Fig. 5(e) and (f). The participants had external rotation on both shoulder joints when walking with and without walking aids. When the participants walked independently, the average external rotation angles of the left and right shoulder joints in the gait cycle were 14.71° and 21.07°, respectively. When using the wheeled walker, the average external rotation angles of the left and right shoulder joints were 16.82° and 18.19°, respectively. When using the walking frame, the external rotation angles of the shoulder joints on both sides first increased and then decreased in the support period. The peak external rotation angle of the left side was 34.29°, and 32.37° of the right side, and separately decreased to 18.56° and 13.30° at the end of the support period. Since the start of the swing period, the external rotation angle of the shoulder joints on both sides increased gradually again.

### Range of motion in the shoulder joints

**Fig. 6:**
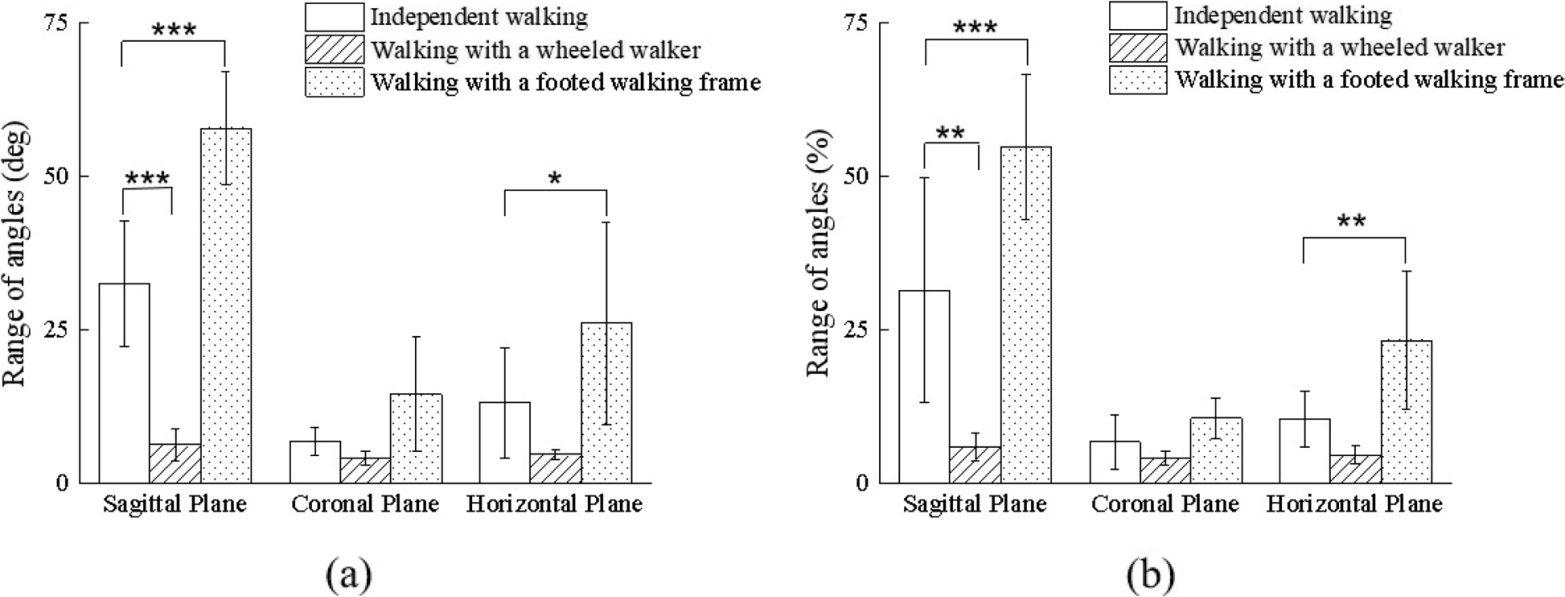
Range of motion of the shoulder joints. **a** Left shoulder joint. **b** Right shoulder joint. Statistical significance: * *p* < 0.05, ** *p* < 0.01, *** *p* < 0.001.

The range of motion in the shoulder joints of the participants in different tasks were shown in Fig. 6. Compared with walking independently, the rotational range of motion in the shoulder joint was significantly decreased in the sagittal plane by using the wheeled walker. Similarly, the rotational range of motion in the shoulder joint was significantly increased in the sagittal and horizontal planes when the participants walked with the walking frame.

## Discussion

Walking aids are important rehabilitation devices that assist older adults to perform daily activities. When the older individuals walking with a aid, the upper limbs compensate for the lower limb functions and reduce the load on the lower limbs, which is of great significance for improving walking ability, satisfying their social needs, and reducing the burden of social management [3, 25–28]. However, the significant changes in the gait parameters of older individuals are caused by the use of walking aids [29]. It is due to the lag of the lower limbs’ motion as a result of walking aids supporting the human body weight and restricting forward when the user pushes or lifts walking aids, which is consistent with the conclusion of Pardo et al. [30].

The pelvis is not only the driving structure of the lower extremities but also the foundation of the upper body during walking. Walking can cause 13.6° forward tilt of the pelvis [24, 31], which is similar to the pelvic anteversion angle in the sagittal plane obtained in this experiment when the participants walked independently. Because of the pelvic anteversion angle increased when the participants used the wheeled walker or the walking frame, the upper limbs required a greater rotational angle to maintain balance. The use of walking aids changes the normal walking pattern, which has a certain impact on the upper limb musculoskeletal system [32]. Therefore, this experiment further studied the effects of different configurations of walking aids on the motion characteristics of the shoulder joints of older individuals by quantitative analysis.

During walking with the wheeled walker, the flexion angle of the shoulder joint in the sagittal plane became larger in pace with the increases in pelvic anteversion angle. Furthermore, the distance between the body and the wheeled walker was relatively stable, so the range of motion of the shoulder joint in the sagittal plane was significantly reduced, which would result in a high flexion angle with narrow range of motion of the joint for a long time. It has been reported that absence or decrease of the joint range of motion of both upper extremities, particularly the shoulder joint, will lead to joint stiffness of upper extremity [14]. The joint stiffness of upper extremity may increase the joint stress under daily activity loads [33]. Therefore, long-term use of wheeled walker is prone to secondary shoulder dysfunction, such as lack or stiffness of the shoulder joint motion, shoulder pain, joint contracture, and osteoporosis [34].

When walking with the footed walking frame, the participant and the walking frame were the furthest apart and the pelvic anteversion angle reached approximately twice that of independent walking in the end of double support period. And the upper limbs moved from extension to the maximum flexion in the sagittal plane, and the external rotation angle decreases in the horizontal plane. Compared with independent walking, the range of motion of the shoulder joint was significantly increased in the sagittal and horizontal planes. Excessive shoulder motion will cause repetitive stress on the shoulder joint and add the load on the muscles around the shoulder joint. Long-term use of a walking frame will also cause cumulative injuries, such as internal wear, rotator cuff injury, and peripheral bursitis [35, 36].

### Limitation

There still are few factors need to be taken into consideration. There may be some differences in physical function between healthy older individuals and the dyskinesia ones. However, the degree of physical decline in the older persons is personalized and difficult to be controlled. To decrease the influence of individuation, six healthy participants without any motion abnormalities were invited. Although data consistency can be observed, the sample size is relatively small. In future, classification research will be conducted based on the different dyskinesias of older individuals with a larger number of participants. In addition, this initial study focused on the motion characteristics of the shoulder joints. Inverse dynamic calculation, and finite element modeling and simulation analysis will be conducted to further evaluate the stress changes in the shoulder joint when the older persons walking with different configurations of walking aids and whether these changes are clinically meaningful.

## Conclusion

This paper explores the effects of different configurations of walking frames on the motion characteristics of the shoulder joints of the older individuals. By comparisons, it was found that the use of walking frames significantly affects the motion of the shoulder joints of the older individuals. The use of a wheeled walker increases the flexion angle of the shoulder joint in the sagittal plane, but the range of motion is significantly reduced, which may lead to secondary shoulder dysfunction. Using a walking frame increases the range of motion of the joint significantly in the sagittal and horizontal planes, which will cause cumulative damage to the shoulder joint and induce musculoskeletal diseases of the shoulder joints. Therefore, the motion characteristics of the upper limbs should be considered when selecting a walking frame to ensure that it fits the functional requirements and physical capabilities of users. This study provides a reference for determining walking aids for older people.

## Data Availability

All data are available upon reasonable request to the corresponding authors Chunjing Tao (chunjingtao@buaa.edu.cn) and Yubo Fan (yubofan@buaa.edu.cn).
